# N-cadherin induces partial differentiation of cholinergic presynaptic terminals in heterologous cultures of brainstem neurons and CHO cells

**DOI:** 10.3389/fnsyn.2012.00006

**Published:** 2012-12-05

**Authors:** Richard J. Flannery, Juan L. Brusés

**Affiliations:** ^1^Department of Anatomy and Cell Biology, University of Kansas School of MedicineKansas City, KS, USA; ^2^Department of Psychiatry and Behavioral Sciences, University of Kansas School of MedicineKansas City, KS, USA

**Keywords:** synapse formation, cell adhesion molecules, cadherin, p120-catenin, cholinergic neurons, neuronal nicotinic acetylcholine receptors, synaptogenic proteins, synaptic vesicle clustering

## Abstract

N-cadherin is a calcium-sensitive cell adhesion molecule commonly expressed at synaptic junctions and contributes to formation and maturation of synaptic contacts. This study used heterologous cell cultures of brainstem cholinergic neurons and transfected Chinese Hamster Ovary (CHO) cells to examine whether N-cadherin is sufficient to induce differentiation of cholinergic presynaptic terminals. Brainstem nuclei isolated from transgenic mice expressing enhanced green fluorescent protein (EGFP) under the control of choline acetyltransferase (ChAT) transcriptional regulatory elements (ChAT^BAC^EGFP) were cultured as tissue explants for 5 days and cocultured with transfected CHO cells for an additional 2 days. Immunostaining for synaptic vesicle proteins SV2 and synapsin I revealed a ~3-fold increase in the area of SV2 immunolabeling over N-cadherin expressing CHO cells, and this effect was enhanced by coexpression of p120-catenin. Synapsin I immunolabeling per axon length was also increased on N-cadherin expressing CHO cells but required coexpression of p120-catenin. To determine whether N-cadherin induces formation of neurotransmitter release sites, whole-cell voltage-clamp recordings of CHO cells expressing α3 and β4 nicotinic acetylcholine receptor (nAChR) subunits in contact with cholinergic axons were used to monitor excitatory postsynaptic potentials (EPSPs) and miniature EPSPs (mEPSPs). EPSPs and mEPSPs were not detected in both, control and in N-cadherin expressing CHO cells in the absence or presence of tetrodotoxin (TTX). These results indicate that expression of N-cadherin in non-neuronal cells is sufficient to initiate differentiation of presynaptic cholinergic terminals by inducing accumulation of synaptic vesicles; however, development of readily detectable mature cholinergic release sites and/or clustering of postsynaptic nAChR may require expression of additional synaptogenic proteins.

## Introduction

Cell adhesion molecules localized at developing synapses mediate homo and heterotypic interactions between pre and postsynaptic membranes. These trans-synaptic interactions can initiate synapse formation and promote synaptic maturation by providing adhesive support between opposing cell membranes, and by regulating intracellular signaling leading to the assembly of pre and postsynaptic machineries (Dalva et al., 2007; Benson and Huntley, 2011).

N-cadherin, a member of the cadherin superfamily of cell adhesion molecules, is a type I cadherin that mediates calcium-sensitive homotypic binding between apposed cell membranes, and regulates cytoskeletal dynamics through interactions with β-catenin and p120-catenin (Hatta et al., 1988; Nollet et al., 2000; Gumbiner, 2005; Pokutta and Weis, 2007). N-cadherin is expressed at both, glutamatergic and cholinergic synaptic contacts from the early stages of synapse formation, where it is believed to promote synapse development (Beesley et al., 1995; Yamagata et al., 1995; Fannon and Colman, 1996; Uchida et al., 1996; Benson and Tanaka, 1998; Huntley and Benson, 1999; Squitti et al., 1999; Brusés, 2000; Phillips et al., 2001; Rubio et al., 2005). Blockade of N-cadherin binding by gene ablation, mRNA knockdown, or by application of function blocking antibodies in cultured cells results in formation of smaller synaptic contacts with poorly differentiated synaptic terminals, reduced synaptic vesicle recycling, and decreased short-term synaptic plasticity (Bozdagi et al., 2004; Jungling et al., 2006; Stan et al., 2010; Aiga et al., 2011). In contrast, repetitive electrical stimulation of hippocampal neurons enhances the adhesive properties of N-cadherin and induces its recruitment to synaptic contacts, which is required for long-term potentiation (Tang et al., 1998; Bozdagi et al., 2000; Tanaka et al., 2000; Huntley et al., 2002). In addition, N-cadherin trans-synaptically regulates transmitter release in cholinergic neurons through a mechanism modulated by SAP97 (Neff et al., 2009). This evidence indicates that N-cadherin homotypic interaction across the synaptic cleft contributes to synapse development by stabilizing the contact between synaptic membranes and recruiting components of the synaptic complex. However, analysis of N-cadherin synaptogenic activity in the absence of other synaptogenic proteins has failed to demonstrate the induction of presynaptic differentiation in cultured glutamatergic neurons (Scheiffele et al., 2000; Linhoff et al., 2009; Stan et al., 2010; Aiga et al., 2011).

This study used an *in vitro* assay for synapse formation to determine whether N-cadherin is sufficient to induce presynaptic terminal differentiation in cultured primary cholinergic neurons. This assay involves the heterologous expression in non-neuronal cells of presumptive synaptogenic proteins together with ligand-activated channels to detect neurotransmitter release (Biederer and Scheiffele, 2007). This *in vitro* synapse formation assay has been used to identify several synaptogenic proteins, including neuroligins, leucine-rich repeat transmembrane proteins (LRRTMs), and synaptic cell adhesion molecules (SynCAMs) using glutamatergic neurons cocultured with transfected human embryonic kidney (HEK) 293 cells or COS-7 cells as postsynaptic elements (Scheiffele et al., 2000; Biederer et al., 2002; Fu et al., 2003; Linhoff et al., 2009). The present study used cholinergic brainstem neurons cocultured with Chinese Hamster Ovary (CHO) cells transfected with N-cadherin and neuronal nicotinic acetylcholine receptor (nAChR) subunits α3 and β4. Immunostaining of synaptic vesicle proteins and electrophysiological assessment of acetylcholine release from contacting axons were used to examine whether N-cadherin promotes differentiation of presynaptic cholinergic terminals.

## Materials and methods

### Animals and neuronal cultures

Cholinergic brainstem nuclei were isolated from embryonic day (E) 16 homozygous transgenic mice expressing enhanced green fluorescent protein (EGFP) under the control of choline acetyltransferase (ChAT) transcriptional regulatory elements (ChAT^BAC^EGFP), which direct EGFP expression during development in central and peripheral cholinergic neurons including cell bodies and processes (Tallini et al., 2006). Homozygous animals [strain B6.RG-Tg(RP23-268L19-EGFP)2Mik/j] were obtained from The Jackson Laboratory (Bar Harbor, ME) and bred in house. Mating was confirmed by the presence of a vaginal plug and was considered E0. Pregnant mice were anesthetized with isoflurane vapors (Isoflo, Abbott, Abbott Park, IL, USA), sacrificed by cervical dislocation, and the embryos were surgically removed and placed in a dissecting dish containing ice-cold Tyrode's solution (NaCl, 134 mM; KCl, 3 mM, NaHCO_3_, 20 mM; MgCl_2_, 1 mM; and CaCl_2_, 3 mM). The embryos were decapitated, the brains removed, and cholinergic nuclei in the brainstem were identified by EGFP expression using an MZ165FC Leica stereomicroscope (Leica, Wetzlar, Germany) equipped with fluorescence illumination, filters for visualizing GFP fluorescence, and a Zeiss AxioCam HRm digital camera (Zeiss, Oberkochen, Germany). Two types of nuclei were dissected, one located close to the dorsal surface and adjacent to the midline of the brainstem corresponding to the location of preganglionic parasympathetic nuclei, and the other located on the ventrolateral quadrant of the brainstem corresponding to the location of the facial motor nucleus according to the Allen Brain Mouse Atlas. The isolated nuclei were collected in ice-cold Neurobasal cell culture medium (Gibco, Invitrogen, Carlsbad, CA). The tissue was cut in pieces of ~1 mm long and placed as tissue explants on poly-L-ornithine (Sigma, St. Louis, MO) and laminin (Invitrogen) treated glass coverslips and cultured in Neurobasal cell culture medium (Gibco) supplemented with 10% fetal bovine serum (FBS)[American Type Culture Collection (ATCC), Manassas, VA], 2 mM GlutaMAX (Gibco), 2% B27 supplement (Gibco), 5 nM NGF (BD Biosciences, Franklin Lakes, NJ), 10 U/ml of penicillin-G, and 10 μg/ml of streptomycin (Gibco). Twenty-four hours after plating, cytosine arabinoside (2 μM) (Sigma) was added to the culture medium. Tissue cultures were kept in a 5% CO_2_ atmosphere in a water-jacketed incubator at 37°C. Animal maintenance and manipulation procedures were approved by the Institutional Animal Care and Use Committee (IACUC) of the University of Kansas School of Medicine.

### Cell lines and western blot analysis

CHO-K1 cells (ATCC CCL-61) were cultured in T-25 plastic bottles in high-glucose Dulbecco's Modified Eagles Medium (DMEM) supplemented with 2 mM L-glutamine and 10% FBS. Cells were trypsinized and replated in 24-well-plastic tissue culture plates and transfected 24 h later with the desired combination of expression vectors using FuGENE Transfection Reagent (Roche, Mannheim, Germany) or Lipofectamine 2000 (Invitrogen) according to the manufacturers' instructions. Twenty-four hours after transfection, CHO cells were replated with 5-days-old brainstem tissue explants and cocultured for an additional 2 days. HEK293 cells (ATCC CRL-1573), COS-7 cells (ATCC CRL-1651), and L cells (CCL-1 NCTC Clone 929) were cultured following ATCC recommendations. For Western blot analysis, cell lines grown in plastic tissue culture dishes were placed on ice, rinsed with ice-cold Dulbecco's phosphate buffered saline (D-PBS) containing 1 mM CaCl_2_ and 0.5 mM MgCl_2_ (Gibco), and lysed in protein extraction buffer [25 mM HEPES pH 7.2, 150 mM NaCl, 1% NP40, EDTA-free protein inhibitor cocktail (Roche, Indianapolis, IN, USA), and 10 mM NaF 1 mM sodium orthovanadate] by pipetting and sonication on ice (10 s, 2 W). Cell lysates were cleared by centrifugation at 14,000 RPM in an Eppendorf (Hamburg, Germany) microfuge at 4°C. The supernatants were transferred to a clean tube and an aliquot removed to determine protein concentration using the BCA protein assay (Pierce, Thermo Scientific, Rockford, IL, USA). Equal amounts of total proteins from each sample were separated by 10% SDS-PAGE, and proteins electrotransferred to a polyvinylidine difluoride (PVDF) membrane. Unspecific binding sites were blocked by incubation for 30 min at room temperature in blocking solution containing 5% non-fat dry milk (Bio-Rad, Hercules, CA, USA) and 0.1% of Tween-20 in Tris-buffered saline (TBS-T). The PVDF membranes were immunoblotted with N-cadherin (32/N-cadherin, BD Biosciences, San Jose, CA, USA) or E-cadherin (BD Biosciences 36/E-cadherin) antibodies, washed, incubated with anti mouse HRP-conjugated secondary antibodies (Jackson ImmunoResearch), and developed by chemoluminescence (GE Healthcare, Fairfield, CT, USA). Membranes were incubated in stripping solution (100 mM β-mercaptoethanol, 2% SDS, 62.5 mM Tris HCl pH 6.7) for 30 min at 50°C, blocked, immunoblotted with β-tubulin mab3408 antibodies (Chemicon, EMD Millipore Corporation, Billerica, MA, USA) and with anti mouse HRP-conjugated secondary antibodies, and developed by chemoluminescence.

### Expression vectors and DNA constructs

Neuronal nAChR subunits α3 and β4 cloned into a pcDNA3.1 expression vector (Invitrogen) were a gift from Dr. J. Stitzel. To generate α3 and β4 nAChR subunit C-terminally fused to a Myc-tag epitope (α3-Myc nAChR and β4-Myc nAChR, respectively), the receptor subunit coding sequences excluding the stop codon were PCR amplified and the products cloned into a 6×-Myc-tag N-terminal tagging vector that was generated by replacing the EGFP coding sequence from the pEGFP-N1 vector (Clontech, Palo Alto, CA) with six repeats of the Myc-tag epitope sequence (MEQKLISEEDLNE). A pcDNA3.1 plasmid carrying the red fluorescent protein TagRFP-T (Shaner et al., 2008) was a gift from Dr. R. W. Tsien. N-cadherin cDNA was obtained from Open BioSystems (Thermo Scientific, Huntsville, AL) and p120-catenin cDNA was a gift from Dr. A. Reynolds. N-cadherin and p120-catenin C-terminally fused to EGFP were constructed by cloning the protein coding sequence without the stop codon into a pEGFP-N1 vector. An expression vector (pcDNA3.1) carrying EGFP with a myristoylation site was used to label the cell membrane. All sequences were verified by sequencing analyses. Plasmid DNA used for transfection was generated using endotoxin free Maxiprep Kits (Qiagen, Valencia, CA).

### Immunocytochemistry and image analysis

Cultured cells were placed on ice, rinsed with ice-cold D-PBS, fixed with 4% paraformaldehyde in D-PBS for 20 min at room temperature, washed with D-PBS, blocked with 5% donkey serum (Jackson ImmunoResearch, West Grove, PA) in D-PBS, and α3-Myc and β4-Myc nAChR were cell surface labeled with a monoclonal antibody against the Myc-tag epitope (clone 9B11) (Cell Signaling Technologies, Danvers, MA) (1:2000 dilution) at room temperature for 1 h. The antibody was washed with D-PBS and cells incubated with an anti mouse IgG Cy3-conjugated secondary antibody (Jackson ImmunoResearch), washed, and mounted with Prolong Gold (Invitrogen). To detect N-cadherin and p120-catenin, cells were fixed in 4% paraformaldehyde, blocked and permeabilized with 5% donkey serum containing 0.2% Triton X-100 (Sigma) in D-PBS, and incubated with anti N-cadherin cytoplasmic domain mouse monoclonal antibodies (clone 32/N-cadherin) (BD Bioscience), washed, and incubated with an anti mouse IgG Cy3-conjugated secondary antibody (Jackson ImmunoResearch). p120-catenin was immunodetected with a mouse monoclonal antibody (Invitrogen, 6H11). Synaptic vesicles were immunodetected with a mouse monoclonal antibody against the synaptic vesicle protein 2 (SV2) (Buckley and Kelly, 1985) [Developmental Studies Hybridoma Bank (DSHB), Iowa City, IL], and with a rabbit polyclonal antibody against synapsin I (Calbiochem, EMD, Gibbstown, NJ).

Brainstem cholinergic explants were cultured for 5 days as described above, and transfected CHO cells were replated with tissue explants and cocultured for 2 additional days. The cocultures were fixed and immunostained with SV2 or synapsin I antibodies, mounted, and imaged with a Nikon C1-SI confocal microscope (Nikon Instruments, Japan) mounted on a Nikon Eclipse 90i up-right microscope, and a Plan Apo 60X/1.4 Nikon oil immersion lens. Optical sections were collected at 1-μm intervals independently for each fluorophore using Nikon EZ-C1 software. The stacks of images were rendered by maximal projection to a single plane and analyzed with MetaMorph image analysis software (Universal Imaging Corp, Dowingtown, PA). Distances were calibrated, each channel was separated, and background fluorescence eliminated by setting a low threshold level of detection. To analyze synaptic vesicle density with SV2 immunodetection, a region of interest was created over EGFP expressing CHO cells and the total area (μm^2^) and average gray value [arbitrary units (AU)] of SV2 immunofluorescence was calculated using the integrated morphometric analysis tool, and normalized to 1000 μ m^2^ of cell surface. Average gray value is the average of the pixel gray scale values contained in an object, which is directly proportional to the overall brightness of the object. To determine the density of synaptic vesicles per axon length, the length (μm) of neurites in contact with transfected CHO cells was measured by hand using MetaMorph software, the pixel area and total gray value (AU) of synapsin I immunofluorescence were calculated and normalized to axon length (μm). Total gray value is the sum of the gray scale values for all pixels contained in the object and serves as an indication of the total amount of fluorescent signal from the object. Values were logged into Excel software (Microsoft, Seattle, WA) and used to calculate the mean and the standard error of the mean (SEM). To assess N-cadherin expression levels, CHO cells were transfected with N-cadherin-EGFP or with N-cadherin-EGFP and p120-catenin-EGFP, fixed 48 h later, and immunostained with anti N-cadherin antibodies as described above. Confocal sections at the level of the cell membrane were acquired at 0.25-μm intervals with a Plan Apo 60X/1.4 Nikon oil immersion lens using the same scanning settings in all cells. The 1.5-μm thick stacks of images were rendered to a single plane, and total pixel area of N-cadherin immunolabeling was measured in each cell in a 25-μm^2^ region using MetaMorph software. One Way ANOVA analysis and *post-hoc* Bonferroni tests were carried out using SigmaStat software (Systat Software, Inc. San Jose, CA). The two-tailed Student's *T*-test was carried out in Excel software.

### Electrophysiology

Forty-eight hours after adding transfected CHO cells to 5 days old brainstem cholinergic tissue explants, a coverslip of cocultured cells was placed in a recording chamber containing extracellular recording solution (NaCl, 150 mM; KCl, 5 mM; CaCl_2_, 2.5 mM, HEPES, 10 mM; and glucose, 150 mM) and mounted on an upright Zeiss Axioskop 2FS Plus microscope. Cells were observed with a Zeiss Achroplan 40X/NA0.8 water immersion lens under differential interference contrast (DIC) and fluorescence light illumination. TagRFP-T-expressing CHO cells intersected by at least one EGFP-expressing axon from the brainstem explant were selected for recording. Three to six MΩ pipette electrodes were filled with intracellular solution (CsCl, 140 mM; HEPES, 10 mM; EGTA, 0.6 mM; and MgATP, 2 mM). After a seal of ≥1 GΩ was obtained, cells were voltage-clamped in whole-cell mode and maintained at −70 mV using a Multiclamp 700A amplifier (Axon Instruments, Sunnyvale, CA), connected to a Digi-Data 1322A analogue-to-digital converter (Digi-Data, Broomfield, CO). Acetylcholine (Sigma) was applied using a 1–2 μm tip diameter glass pipette containing 5 mM acetylcholine in extracellular solution after 1 min of continuous recording. Light positive pressure using a syringe was sufficient to deliver acetylcholine through the pipette. Recordings were continued for ~5 min to monitor synaptic events. For experiments using tetrodotoxin (TTX) (Sigma), extracellular solution with 1 μM TTX was perfused into the recording chamber. Solutions were held at room temperature (20–22°C) during recordings. Excitatory postsynaptic potentials (EPSPs) were analyzed off-line using P-clamp software with an amplitude threshold cutoff of 20 pA. Individual events were analyzed for duration, amplitude and tau, and for responses with biphasic decay, the Levenberg-Marquardt method gave an accurate fit to the standard bi-exponential function (options in P-clamp software) based on visual appearance.

## Results

**Figure 1:**
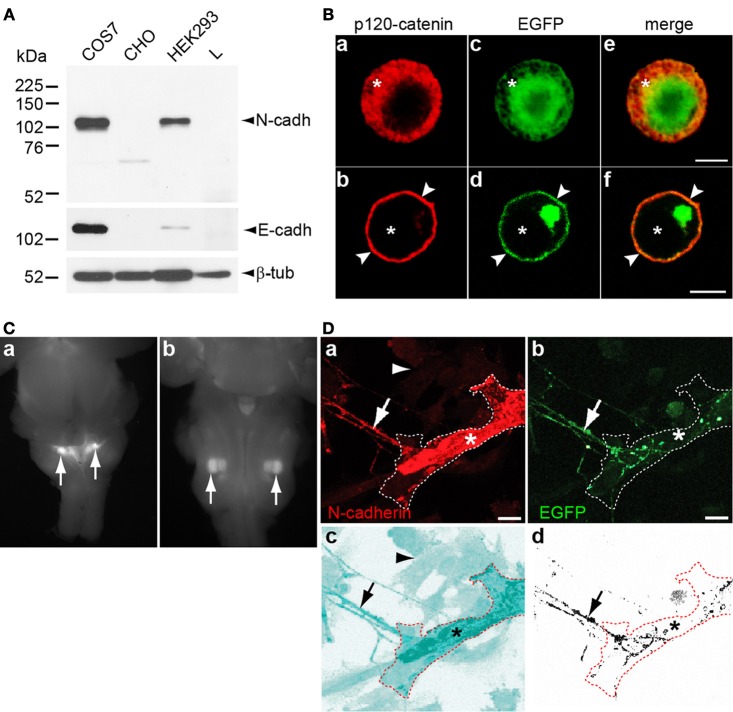
**(A)** Cultured COS-7, CHO, HEK293, and L cells were lysed and analyzed by Western blot with antibodies against N-cadherin, E-cadherin, and β-tubulin. N-cadherin was detected in COS-7 and HEK293 cells, while E-cadherin detected in COS-7 cells and at very low levels in HEK293 cells. CHO and L cells were devoid of both cadherins. **(B)** CHO cells transfected with EGFP (**a**, **c**, and **e**) or with N-cadherin-EGFP (**b**, **d**, and **f**) were fixed 48 h after transfection and immunolabeled with anti p120-catenin antibodies and Cy3-conjugated secondary antibodies (red). In EGFP transfected cells, p120-catenin was localized to the cytosol (asterisks) while expression of N-cadherin shifted the subcellular localization of p120-catenin to the cell membrane (arrowheads in **b**, **d**, and **f**). **(C)** Dorsal **(a)** and ventral **(b)** views of E16 ChAT^BAC^EGFP mouse brainstem showing EGFP expressing nuclei in the dorsomedial region **(a)** and in the ventrolateral quadrant **(b)** corresponding to the localization of preganglionic parasympathetic neurons [**(a)** arrows], and of the facial motor nucleus [**(b)** arrows], respectively. **(D)** N-cadherin transfected CHO cells were cocultured with 5 days old brainstem tissue explants of cholinergic nuclei expressing EGFP for 2 days, fixed, and immunostained for N-cadherin. **(a,b)** N-cadherin (red) expressing CHO cell (asterisks) interacting with a EGFP and N-cadherin expressing cholinergic axon (arrow). The contour of the transfected CHO cell is delineated with a white dashed line. **(c)** The micrograph shown in panel **(a)** was processed with the inversion tool of Adobe Photoshop to facilitate the visualization of the N-cadherin transfected CHO cell (asterisk) and non-transfected CHO cells [arrowhead in **(a)** and **(c)**] that do not express N-cadherin. The contour of the N-cadherin expressing CHO is delineated with a dashed red line and the arrow points to the contacting axon. **(d)** The EGFP signal from panel **(b)** was selected and copied to a new panel with white background to visualize the axons (arrow) from the brainstem explant growing on top of the N-cadherin expressing CHO cell delineated with a dashed red line (asterisk). Scale bars in **(B)**, 5 μm; in **(D)**, 10 μm.

To examine whether N-cadherin induces presynaptic differentiation in cholinergic axons, an *in vitro* assay of synapse formation was used. This assay consists of the coculturing of primary neurons serving as presynaptic elements, with a non-neuronal cell line incapable of triggering presynaptic differentiation. Expression of a synaptogenic protein that is not endogenously expressed by the non-neuronal cell, transforms the non-neuronal cell type into a suitable postsynaptic element capable of inducing presynaptic differentiation on contacting neurites by promoting the accumulation of synaptic vesicles and the development of an active zone (Biederer and Scheiffele, 2007). To select a suitable cell line for the synapse formation assay, N-cadherin expression was examined by Western blot analysis in cell types commonly used in heterologous synapse formation assays. COS-7, HEK293, CHO, and L cell lysates immunoblotted with N-cadherin antibodies showed that COS-7 and HEK293 cells both endogenously expressed N-cadherin, while CHO and L cells did not (Figure 1A). E-cadherin was also detected in COS-7 cells while CHO and L cells were both devoid of E-cadherin expression, and very little amounts were detected in HEK293 cells. To examine whether any other type I or type II cadherin was expressed in CHO cells, cultured CHO cells were immunolabeled with anti p120-catenin antibodies. p120-catenin binds to the membrane proximal region of the cytoplasmic domain of type I and type II cadherins, and no other cell membrane protein is known to bind p120-catenin (Reynolds et al., 1996; Anastasiadis and Reynolds, 2000). In CHO cells transfected with EGFP, p120-catenin localized to the cytosol, while in cells transfected with N-cadherin-EGFP, the subcellular localization of p120-catenin shifted to the cell membrane (Figure 1B) (Rubio et al., 2005). These results indicate that CHO cells do not express classical cadherins. Thus, brainstem cholinergic neurons cocultured with transfected CHO cells were used for a heterologous synapse formation assay to examine the role of N-cadherin in the differentiation of cholinergic presynaptic terminals. Only CHO cells were used for the synapse formation assay because L cells showed very low transfection efficiency.

Brainstem cholinergic neurons have their cell bodies in parasympathetic and motor nuclei, and send axons to synapse with peripheral ganglionic neurons and muscle cells, respectively. EGFP expressing cholinergic nuclei were isolated from E16 transgenic mice expressing EGFP under the control of the ChAT transcriptional regulatory elements (ChAT^BAC^EGFP) (Tallini et al., 2006) and cultured as tissue explants. The dissected cholinergic nuclei were located in the dorsomedial region and in the ventrolateral quadrant of the brainstem corresponding to the localization of parasympathetic preganglionic (Figure 1Ca) and facial motor neurons (Figure 1Cb), respectively. Axons extending out of the explants served as presynaptic elements for the synapse formation assay. CHO cells transfected with N-cadherin and cocultured with cholinergic neurons were immunostained for N-cadherin and revealed that both cultured cholinergic axons and transfected CHO cells expressed N-cadherin (Figure 1D).

**Figure 2:**
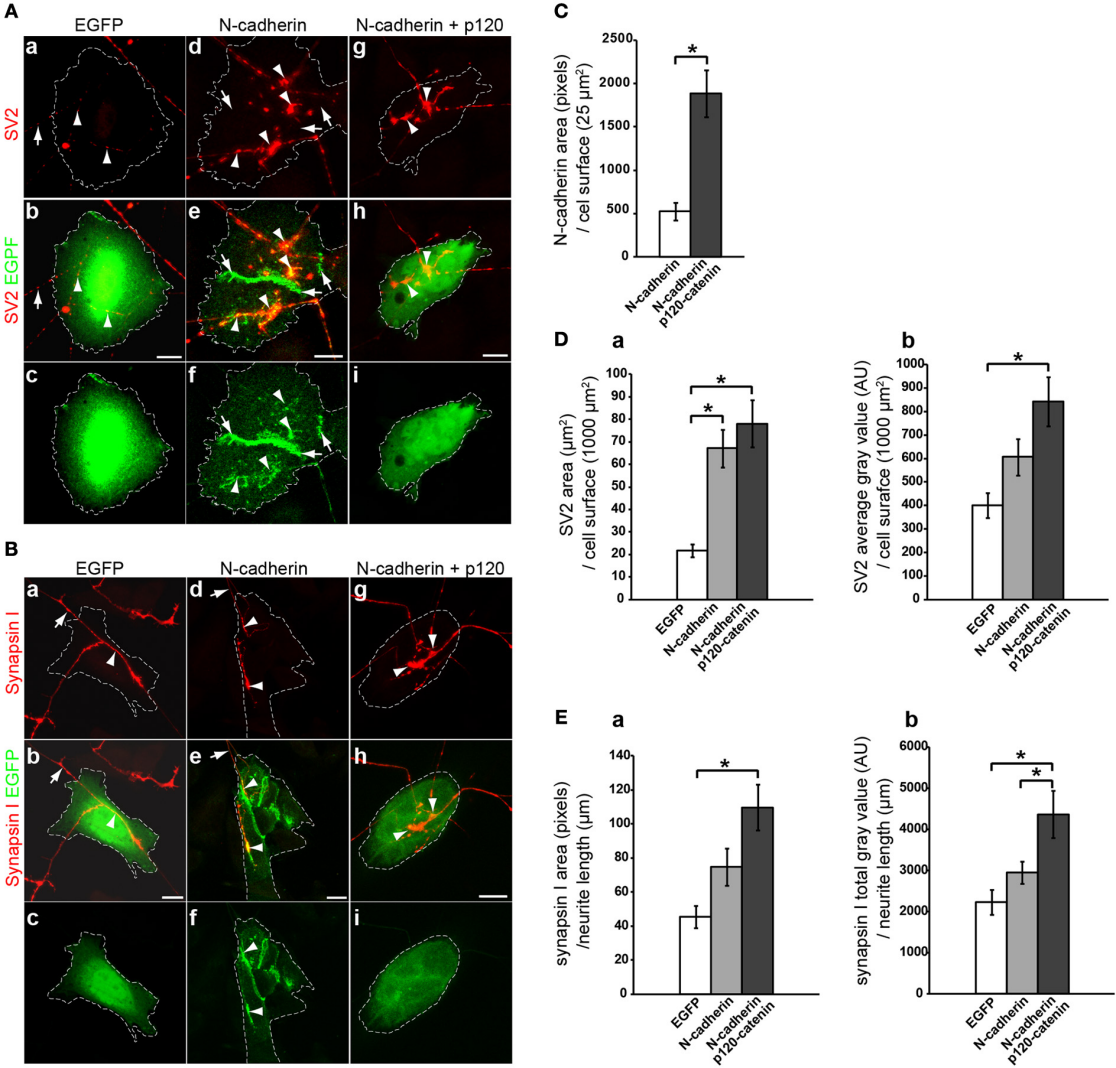
**Analysis of N-cadherin-mediated effect on synaptic vesicle accumulation in cholinergic neurons.** CHO cells transfected with EGFP, N-cadherin-EGFP, or N-cadherin-EGFP and p120-catenin-EGFP were plated on 5 days old brainstem cholinergic nuclei explants and cocultured for 2 days. Tissue cultures were fixed and immunostained with SV2 **(A)** or synapsin I **(B)** antibodies. **(A,B)** Confocal images of CHO cells transfected with EGFP **(a,b,c)**, N-cadherin-EGFP **(d,e,f)**, or N-cadherin-EGFP and p120-catenin-EGFP **(g,h,i)**. Panels **(a)**, **(d)**, and **(g)**, show SV2 or synapsin I immunostaining (red); panels **(b)**, **(e)**, and **(h)**, show merged images with EGFP fluorescence (green); and panel **(c)**, **(f)**, and **(i)**, show EGFP fluorescence alone. A white dashed line delineates the contour of the transfected CHO cell in each panel. **(C)** N-cadherin expression levels in CHO cells transfected with N-cadherin or cotransfected with N-cadherin and p120-catenin were measured as pixels of N-cadherin immunolabeling over CHO cell surface area (μm^2^) (N-cadherin, *n* = 31 cells; N-cadherin and p120-catenin, *n* = 27 cells). **(D)** Analysis of **(a)** SV2 immunolabeled area (μm^2^) over transfected CHO cells normalized to CHO cell surface area (μm^2^), and **(b)** SV2 average gray value [arbitrary units (AU)] over transfected CHO cells normalized to CHO cell surface area (μm^2^) [EGFP (*n* = 18), N-cadherin (*n* = 24), and N-cadherin and p120-catenin (*n* = 24)]. **(E)** Analysis of **(a)** synapsin I immunolabeled area (pixels) normalized to neurite length (μm) in contact with transfected CHO cells, and **(b)** synapsin I total gray value (AU) per neurite length (μm) in contact with transfected CHO cells [EGFP (*n* = 10), N-cadherin (*n* = 14), and N-cadherin and p120-catenin (*n* = 12)]. **(C)** Student's *T*-test, ^*^p < 0.005. **(D,E)** One-Way ANOVA analysis in **(Da)**, **(Db)**, **(Ea)**, and **(Eb)**, *p* < 0.005, and *post-hoc* Bonferroni pairwise comparisons, ^*^p < 0.05. Scale bars, 10 μm.

To examine the effect of N-cadherin on synaptic vesicle density, 5 days old tissue explants of brainstem cholinergic nuclei were cocultured with CHO cells transfected with EGFP or with N-cadherin for an additional 2 days. The cocultures were then fixed and immunolabeled with SV2 or synapsin I antibodies to detect synaptic vesicles in cholinergic axons contacting transfected CHO cells. Cells were imaged under confocal microscopy and the stacks of images were rendered to a single plane and analyzed with MetaMorph software. The effect of N-cadherin on axon growth and synaptic vesicle accumulation was assessed by measuring SV2 immunofluorescence area and average gray value on axons in contact with transfected CHO cells normalized to 1000 μm^2^ of CHO cell surface area (Figures 2A,D). A ~3-fold increase in the area of SV2 immunofluorescence (EGFP 21.7 ± 3.0 μm^2^, N-cadherin 67.1 ± 8.3 μm^2^) (Figure 2Da) and a ~1.5-fold increase in SV2 average gray value (EGFP 400.9 ± 52.1 AU and N-cadherin 606.8 ± 77.9 AU) (Figure 2Db) per CHO cell surface area, were observed in N-cadherin expressing CHO cells as compared to axons in contact with CHO cells transfected only with an EGFP expression vector.

However, only the increase in SV2 pixel area on N-cadherin expressing CHO cells showed a statistically significant difference with control cells (Figure 2Da), while the increase observed in average gray value was not significant (Figure 2Db), suggesting that the effect of N-cadherin expression detected by SV2 immunostaining was more prominent on axon growth than on the accumulation of synaptic vesicles. Finally, N-cadherin accumulated at the sites of contact between axons and N-cadherin expressing CHO cells, suggesting that the increase in axon growth was mediated by N-cadherin homotypic interactions (Figure 2A).

Binding of p120-catenin to the membrane proximal region of type I cadherins cytoplasmic domain regulates cadherin turnover by masking an endocytotic signal (Ireton et al., 2002; Davis et al., 2003; Nanes et al., 2012). Knockdown of p120-catenin disrupts the stability of intercellular junctions due to a decrease in cadherin expression, while overexpression of p120-catenin increases cell surface levels of cadherins expression (Davis et al., 2003). Therefore, if the effect on SV2 accumulation observed on N-cadherin transfected CHO cells was related to N-cadherin expression, it was expected that an increase in N-cadherin stability by overexpressing p120-catenin would enhance SV2 accumulation in contacting axons. To determine whether overexpression of p120-catenin leads to an increase in N-cadherin expression levels in CHO cells, CHO cells were transfected with N-cadherin alone or cotransfected with N-cadherin and p120-catenin, and immunolabeled with anti N-cadherin antibodies. Analysis of N-cadherin density showed a ~3.6-fold increase in the levels of N-cadherin expression in cells co-transfected with p120-catenin (522.6 ± 102.1 and 1885.2 ± 268.3 pixels/25 μm^2^ of cells surface area, respectively) (Figure 2C). Cotransfection with p120-catenin also resulted in a statistically significant increase of both, total area and average gray value of SV2 over CHO cells cocultured with cholinergic brain stem explants when compared to untransfected CHO cells (78.1 ± 10.5 μm^2^ and 843.1 ± 104.0 AU) (Figure 2D). These results indicate that coexpression of N-cadherin and p120-catenin in heterologous cells increases the density of synaptic vesicles per cell surface area either by promoting neurite growth or by inducing the accumulation of synaptic vesicles at the site of contact between axons and N-cadherin expressing cells.

To distinguish between these two possibilities, the accumulation of synapsin I immunolabeling per axon length of brainstem cholinergic axons in contact with EGFP or N-cadherin transfected CHO cells was assessed (Figures 2B,E). CHO cells transfected with N-cadherin showed an increase in the total amount of synaptic vesicles per axon length (EGFP 45.3 ± 6.6 μm^2^ and N-cadherin 74.8 ± 10.8 μm^2^) (Figure 2Ea), and a ~1.3-fold increase in synapsin I total gray value (EGFP 2230.1 ± 305.0 AU, N-cadherin 2945.7 ± 269.4 AU) (Figure 2Eb), as compared to CHO cells transfected only with EGFP. However, the increase in synaptic vesicle accumulation mediated by N-cadherin expression was not statistically significant. To examine whether the effect on synaptic vesicle accumulation associated with N-cadherin expression was sensitive to overexpression of p120-catenin, CHO cells cotransfected with both N-cadherin and p120-catenin were cocultured with brainstem cholinergic nuclei explants and synapsin I immunolabeling was analyzed as described above. Cotransfection of p120-catenin with N-cadherin resulted in a further increase in the accumulation of synaptic vesicles per axon length as indicated by the statistically significant increase in both synapsin I immunolabeled area and total gray value (109.8 ± 13.5 μm^2^ and 4358.0 ± 572.7 AU) (Figure 2E). Thus, heterologous expression of N-cadherin in CHO cells promotes axon growth and synaptic vesicle accumulation indicating that N-cadherin is sufficient to induce presynaptic differentiation in axons extending from brainstem cholinergic neurons. However, the effect of N-cadherin on synaptic vesicle accumulation requires overexpression of p120-catenin, suggesting that stabilization of N-cadherin on the cell surface in needed to induce presynaptic differentiation.

**Figure 3:**
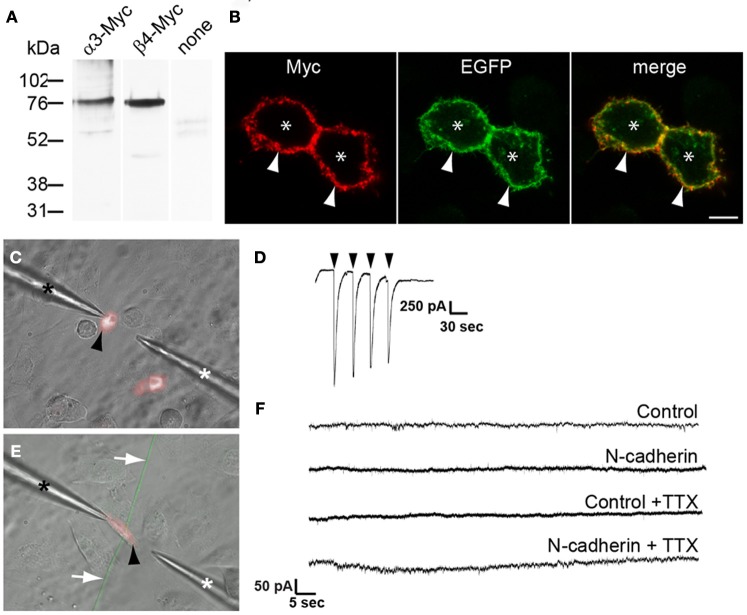
**Electrophysiological analysis of the effect of N-cadherin on α3β4 nAChR response in transfected CHO cells in contact with cholinergic axons from brainstem nuclei explants. (A)** Immunoblot detection with anti Myc-tag antibodies of nAChR subunit expression in cell lysates of CHO cells transfected with α3-Myc, β4-Myc, or untransfected (none). A band of ~76 kDa was detected with both nAChR subunits. **(B)** Confocal images across the center of CHO cells cotransfected with α3-Myc, β4-Myc, and membrane-bound myristoylated EGFP (green), and immunostained with anti Myc-tag antibodies and Cy3-conjugated secondary antibodies (red). Asterisks indicate CHO cell bodies and arrows point to the cell membrane. **(C)** Microphotograph of cultured CHO cells transfected with TagRFP-T and α3β4 nAChR subunits (black arrowhead) in the recording chamber. One pipette was used for patch-clamp recording (black asterisk) and a second pipette was used to deliver acetylcholine (white asterisk). **(D)** Voltage-clamp recording of a CHO cell transfected with α3β4 nAChR subunits and TagRFP-T, and exposed to a solution of 5 mM acetylcholine (arrowheads) confirmed the expression of functional α3β4 nAChRs on the cell surface. **(E)** Representative microphotograph of a TagRFP-T and α3β4 nAChR transfected CHO cell (black arrowhead) used to evaluate formation of neurotransmitter release sites in contacting EGFP-expressing cholinergic axons (white arrows). CHO cells were cotransfected with N-cadherin and cells in which the N-cadherin expression vector was omitted from the transfection mix were used as controls. Expression of α3β4 nAChR was confirmed by eliciting inward currents in response to acetylcholine. One pipette was used for patch-clamp recording (black asterisk) and a second pipette was used to deliver acetylcholine (white asterisk). **(F)** Representative recordings of control and N-cadherin transfected CHO cells in contact with at least one axon extending from a cholinergic explant. No EPSPs were observed in control (*n* = 6) and in N-cadherin (*n* = 11) transfected CHO cells, and no mEPSPs were detected in control (*n* = 7) or N-cadherin (*n* = 12) transfected cells in the presence of TTX. Scale bar in **(B)**, 5 μm.

To determine whether N-cadherin induces formation of cholinergic neurotransmitter release sites, CHO cells transfected with or without N-cadherin were cotransfected with α3 and β4 nAChR subunits, and used to detect acetylcholine release by whole-cell recordings under voltage-clamp configuration. α3β4 nAChR subunits form a heterologous pentameric ligand-activated ion channel, which opens in response to acetylcholine (Conroy et al., 2003; Albuquerque et al., 2009). Channels with α3β4 subunit composition are the most common nAChRs expressed in autonomic neurons and drive cholinergic neurotransmission in autonomic ganglia (Conroy et al., 2003; Albuquerque et al., 2009). Western blot analysis of CHO cells transfected with α3 or β4 receptor subunits C-terminally fused to a Myc-tag epitope (α3-Myc and β4-Myc, respectively) showed expression of both subunits (Figure 3A). Cell surface labeling with an anti Myc-tag antibody of CHO cells cotransfected with α3-Myc and β4-Myc nAChR subunits, showed that nAChRs were expressed on the cell surface indicating that this subunit combination is transported to the cell membrane in CHO cells (Figure 3B). Cotransfection of α3β4 nAChR subunits with a TagRFP-T expression vector was used to identify transfected cells under fluorescence light illumination in the recording chamber (Figure 3C). Pressure application of acetylcholine (5 mM) applied through a pipette placed in close proximity to a patch-clamped transfected CHO cell (Figure 3C), elicited inward currents ranging from 200 pA to 4 nA, demonstrating that α3β4 nAChRs subunits formed functional ligand-activated ion channels when expressed in CHO cells (Figure 3D).

CHO cells transfected with α3β4 nAChR subunits, TagRFP-T, and with or without N-cadherin, were added to 5 days old brainstem cholinergic nuclei explants and recorded from 2 days later. Whole-cell recordings under voltage-clamp configuration of CHO cells expressing TagRFP-T, responding to exogenous application of acetylcholine, and intersected by at least one cholinergic axon were used to examine the appearance of EPSPs (Figure 3E). EPSPs were not observed in control cells not expressing N-cadherin (*n* = 6) (Figure 3F). Similarly, no EPSPs were observed in the majority of N-cadherin expressing CHO cells cultured with autonomic or motor nuclei (10 out of 11) (Figure 3F). In one N-cadherin transfected CHO cell, eleven consecutive depolarizations with an average amplitude of 250.0 ± 6.0 pA were detected during a 90 s period (data not shown). These events showed a biphasic decay characterized by a broad peak or slow decay followed by a rapid decay, and could be fitted to a bi-exponential function (decay tau_1_ = 42.4 ± 10.4, tau_2_ = 5.2 ± 0.6). However, the amplitude and kinetics of these events did not match typical cholinergic EPSPs (Chen et al., 2001), suggesting that they did not occur in response to presynaptic neurotransmitter release, but the source of these events could not be determined. Finally, spontaneous transmitter release was examined by monitoring miniature EPSPs (mEPSPs) from cholinergic axons in contact with transfected CHO cells in the presence of TTX. mEPSPs were not detected in either control (*n* = 7) or N-cadherin (*n* = 12) transfected CHO cells, indicating that the probability of transmitter release in cultured cholinergic neurons was very low and it was not enhanced by N-cadherin expression (Figure 3F). These results indicate that N-cadherin by itself is not sufficient to induce formation of functionally mature synapses in cultured cholinergic brainstem neurons within 48 h of culture. This may be due to the inability of N-cadherin to promote development of mature neurotransmitter release sites and/or clustering of postsynaptic neurotransmitter receptors within this time frame. It remains to be determined whether coexpression of N-cadherin and p120-catenin promotes the assembly of neurotransmitter release sites.

## Discussion

Analysis of N-cadherin expression in the developing nervous system showed that N-cadherin is localized at glutamatergic and cholinergic synaptic contacts from early stages of synapse development, suggesting that N-cadherin participates in synapse formation (Fannon and Colman, 1996; Uchida et al., 1996; Benson and Tanaka, 1998; Bozdagi et al., 2000; Brusés, 2000, 2006; Rubio et al., 2005). This supposition is supported by the fact that blockade of N-cadherin binding in cultured cells impaired synapse formation and function, indicating that N-cadherin regulates certain aspects of synapse formation and/or maturation (Bozdagi et al., 2004; Jungling et al., 2006; Stan et al., 2010; Aiga et al., 2011). In glutamatergic neurons, N-cadherin contributes to synapse development by recruiting synaptogenic molecules to the nascent synaptic contact (Stan et al., 2010). However, previous studies indicated that overexpression of N-cadherin in non-neuronal cell lines does not promote presynaptic terminal differentiation in glutamatergic neurons (Scheiffele et al., 2000; Linhoff et al., 2009). The present study was aimed at determining whether N-cadherin is sufficient to induce presynaptic differentiation in cholinergic neurons using brainstem tissue explants cocultured with transfected CHO cells as an *in vitro* assay for synapse formation. This study found that heterologous expression of N-cadherin in CHO cells promoted axon growth but failed to induce the accumulation of synaptic vesicles in cholinergic axons unless N-cadherin was cotransfected with p120-catenin. As p120-catenin stabilizes N-cadherin on the cell surface by decreasing its turnover, the results suggest that expression of N-cadherin above a certain threshold level is necessary to induce presynaptic differentiation. In addition, acetylcholine release sites were not detected by electrophysiological assessment of neurotransmitter release in CHO cells expressing α3β4 nAChR and N-cadherin, indicating that N-cadherin expressed in cultured CHO cells is sufficient to initiate certain aspects of cholinergic synaptic differentiation but failed to promote formation of active synaptic contacts within 48 h of culture. As observed with the effect of N-cadherin on synaptic vesicle accumulation, it is possible that overexpression of p120-catenin is required to induce formation of active neurotransmitter release sites; however, whether p120-catenin contributes to the assembly transmitter release sites was not examined.

Acetylcholine release was not detected in both parental and N-cadherin transfected CHO cells cocultured with brainstem cholinergic neurons when recordings were done in the presence of TTX, indicating that the probability of transmitter release in these neurons was very low. The lack of detection of spontaneous transmitter release may be due to an intrinsic property of brainstem cholinergic neurons. In several autonomic ganglia receiving cholinergic input from brainstem neurons there is a low threshold for spike generation (Rang, 1981; Katayama and Nishi, 1984; Wang et al., 2002). A single EPSP from the preganglionic neuron is capable of inducing an action potential in the postganglionic neuron (Wang et al., 2002). Quantal content from these synapses is quite high (~100 vesicles), whereas the frequency of mEPSPs is low (Katayama and Nishi, 1984), and in some cases it is too low to be recorded (Rang, 1981). This may explain why mEPSPs were not detected in transfected CHO cells in contact with cholinergic axons. It is also possible that the amplitude of mEPSPs was below the threshold of detection. In the absence of TTX, inward currents were detected in one N-cadherin transfected CHO cell; however, the kinetics of the responses indicated that the currents were unlikely generated by presynaptic transmitter release. In a separate experiment, EPSPs were detected in HEK293 cells transfected with neuroligin 1 and α3β4 nAChRs in contact with cholinergic axons, indicating that cultured brainstem neurons elicited transmitter release (Richard J. Flannery and Juan L. Brusés, unpublished observations). Thus, the failure to detect transmitter release in N-cadherin expressing CHO cells may be because N-cadherin is not sufficient to induce differentiation of functional transmitter release sites, or because N-cadherin fails to recruit components of the postsynaptic apparatus, including the clustering of nAChR apposed to neurotransmitter release sites. This effect has been observed with thrombospondin 1 on glutamatergic synapses, in which thrombospondin 1 induced differentiation of presynaptic terminals and transmitter release sites, but the formed synapses were silent due the absence of AMPA receptor clusters on the postsynaptic membrane (Christopherson et al., 2005). Thus, N-cadherin may be incapable of recruiting nAChR or may actively promote the dispersion of nAChR from the site of cell-cell contact. Further experiments are required to determine the extent of cholinergic pre and postsynaptic terminal differentiation induced by N-cadherin, and to determine whether cotransfection with p120-catenin is necessary to transform the silent contacts into active synaptic sites.

In glutamate-releasing neurons, synapse formation appears to be a multistep process in which N-cadherin initiates contact between synaptic membranes and recruits neuroligins, which induce synaptic vesicle clustering and formation of an active zone (Stan et al., 2010; Aiga et al., 2011). Overexpression of N-cadherin in HEK293 and COS-7 cells did not promote accumulation of synaptic vesicles in cultured glutamatergic neurons (Scheiffele et al., 2000; Linhoff et al., 2009), suggesting that N-cadherin by itself is incapable of promoting presynaptic differentiation in this neuronal type. In contrast, the present study found that expression of N-cadherin in CHO cells was sufficient to induce axon growth in cholinergic axons as compared to cells that did not express N-cadherin; however, the effect of N-cadherin on synaptic vesicle accumulation required overexpression of p120-catenin. This suggests that either N-cadherin initiates presynaptic terminal differentiation in cholinergic neurons but not in glutamatergic neurons or that overexpression of N-cadherin alone in HEK293 or COS-7 cells is not sufficient to show an increase in presynaptic differentiation above control levels. It remains to be determined whether overexpression of p120-catenin in HEK293 and COS-7 cells induces synaptic vesicle accumulation in glutamatergic neurons. Alternatively, it is possible that differences in the molecular repertoire expressed in each of these cell lines affect the role of N-cadherin in presynaptic differentiation. In any case, the efficiency of N-cadherin in inducing cholinergic synaptic vesicle accumulation *in vitro* is lower than the one observed with neuroligin-1, SynCAM, and LRRTMs in glutamatergic neurons (Scheiffele et al., 2000; Biederer et al., 2002; Linhoff et al., 2009). In addition, N-cadherin-mediated presynaptic differentiation may not generate functionally mature synapses, suggesting that the mechanism whereby N-cadherin drives synapse formation *in vivo* differs from the one used by other synaptogenic molecules.

The fact that cotransfection of N-cadherin with p120-catenin increased the ability of the CHO cells to induce presynaptic differentiation is interesting because p120-catenin binds to N-cadherin in ciliary ganglion neurons at the time cholinergic synapses form *in vivo* (Rubio et al., 2005). In contrast, p120-catenin uncouples from N-cadherin when ganglionic cholinergic synaptic contacts have matured, even though N-cadherin localization at synaptic sites increases (Rubio et al., 2005), suggesting that the increase in synaptogenic activity of CHO cells cotransfected with p120-catenin in cholinergic neurons may not only be due to an increase in N-cadherin surface expression levels. Although binding of p120-catenin to cadherins on the cell membrane decreases cadherins turnover and increases cell surface expression levels (Davis et al., 2003), the coupling of p120-catenin with cadherins can positively or negatively affect cell adhesion (Ireton et al., 2002). Thus, binding of p120-catenin to N-cadherin cytoplasmic domain may cause allosteric changes in N-cadherin extracellular domain that affect downstream events in the presynaptic cell triggered by N-cadherin homotypic binding. Binding of p120-catenin to cadherins in glutamatergic presynaptic terminals contribute to presynaptic differentiation and clustering of synaptic vesicles (Lee et al., 2008), suggesting that coupling of p120-catenin to N-cadherin in postsynaptic cells may increase the activity of N-cadherin in presynaptic differentiation. As p120-catenin regulates RhoA and voltage-activated calcium influx in cholinergic neurons (Anastasiadis et al., 2000; Piccoli et al., 2004; Marrs et al., 2009), this results suggests that coupling and uncoupling of p120-catenin from N-cadherin may serve as a switch that regulates the activity of N-cadherin and downstream signaling pathways during formation and maturation of cholinergic synapses (Brusés, 2006).

In conclusion, this study found that N-cadherin trans-synaptically induces axonal growth and accumulation of synaptic vesicles in brainstem cholinergic neurons cocultured with transfected CHO cells overexpressing p120-catenin. As nascent synaptic sites lack many of the components necessary for efficient synaptic transmission (Cohen-Cory, 2002), these results suggest that N-cadherin is sufficient to induce partial differentiation of synaptic sites in cholinergic neurons but that differentiation of functionally mature synaptic contacts may require more time, the interaction with p120-catenin, or the cooperative action of other synaptogenic proteins.

### Conflict of interest statement

The authors declare that the research was conducted in the absence of any commercial or financial relationships that could be construed as a potential conflict of interest.
